# Lessons learned from the mechanisms of posttraumatic inflammation extrapolated to the inflammatory response in COVID-19: a review

**DOI:** 10.1186/s13037-020-00253-7

**Published:** 2020-07-09

**Authors:** Michel P. J. Teuben, Roman Pfeifer, Henrik Teuber, Leonard L. De Boer, Sascha Halvachizadeh, Alba Shehu, Hans-Christoph Pape

**Affiliations:** 1 Department of Traumatology, University Hospital Zurich, Raemistrasse 100, 8006 Zurich, Switzerland; 2 Harald Tscherne Laboratory for Orthopedic Research, Zurich, Switzerland; 3 Department of Spine- Neuro- and Special orthopedic Surgery, Rhein-Maas Klinikum Würselen, Aachen, Germany; 4 Department of Surgery, Cantonal Hospital Frauenfeld, Frauenfeld, Switzerland; 5 Imperial College London, London, UK; 6 The Francis Crick Institute, London, UK; 7 Department of Trauma and Orthopedic Surgery, Marienhospital, Aachen, Germany

**Keywords:** Covid-19, SARS-CoV-2, Severe trauma, critical care, ARDS, Inflammation

## Abstract

Up to 20% of Severe Acute Respiratory Syndrome Coronavirus 2 (SARS-CoV-2) patients develop severe inflammatory complications with diffuse pulmonary inflammation, reflecting acute respiratory distress syndrome (ARDS). A similar clinical profile occurs in severe trauma cases. This review compares pathophysiological and therapeutic principles of severely injured trauma patients and severe coronavirus disease 2019 (COVID-19).

The development of sequential organ failure in trauma parallels deterioration seen in severe COVID-19. Based on established pathophysiological models in the field of trauma, two complementary pathways of disease progression into severe COVID-19 have been identified. Furthermore, the transition from local contained disease into systemic and remote inflammation has been addressed. More specifically, the traumatology concept of sequential insults (‘hits’) resulting in immune dysregulation, is applied to COVID-19 disease progression modelling. Finally, similarities in post-insult humoral and cellular immune responses to severe trauma and severe COVID-19 are described.

To minimize additional ‘hits’ to COVID-19 patients, we suggest postponing all elective surgery in endemic areas. Based on traumatology experience, we propose that immunoprotective protocols including lung protective ventilation, optimal thrombosis prophylaxis, secondary infection prevention and calculated antibiotic therapy are likely also beneficial in the treatment of SARS-CoV-2 infections. Finally, rising SARS-CoV-2 infection and mortality rates mandate exploration of out-of-the box treatment concepts, including experimental therapies designed for trauma care.

## Background

Originating from the Wuhan Province in China, the novel member of the coronoviridae family named Severe Acute Respiratory Syndrome Coronavirus 2 (SARS-CoV-2) has rapidly developed into a global pandemic [1, 2]. Given the rising infection and mortality rates that have already or may soon overwhelm our critical care infrastructure, out-of-the-box treatment concepts should immediately be explored [3]. Up to 20% of SARS-CoV-2 infected patients develop severe inflammatory complications with diffuse pulmonary inflammation, reflecting acute respiratory distress syndrome (ARDS) [4, 5]. Similarly, a pattern of respiratory failure occurs in a subgroup of severely injured trauma patients [6]. ARDS is a frequent contributing factor towards morbidity and mortality after trauma [7]. Indeed, a recent meta-analysis demonstrates that the incidence of trauma-induced ARDS has not changed over the last few decades or varied between geographic regions [8].

Given their similar clinical profiles, there may be more similarities between severe trauma and severe cases of SARS-CoV-2 than might initially be assumed. In this review, we will consider the notion that severe disease progression in SARS-CoV-2/Corona Virus Disease 2019 (COVID-19) patients mimics disease mechanisms that occur in complicated courses of severe trauma*.*

There is a broad spectrum in clinical courses of both severe trauma and severe illness caused by SARS-CoV-2 infection [4, 9–12]. Most cases present a mild or moderate clinical course and prompt recovery occurs. However, in both conditions, a specific subgroup of about 20% of patients develop a complicated course characterized by an overwhelming inflammatory response resulting in a life-threatening condition and high mortality [4, 10, 13, 14]. In both disease processes, the difference between a mild and severe clinical course depends on the development of sequential organ failure. As in trauma, this cascade typically starts with the development of pulmonary failure: ARDS, sequentially leading to multiple organ dysfunction syndrome (MODS) and death [6, 14–17]. Documented mortality rates among intensive care unit (ICU)-admitted COVID-19 patients have to date ranged from 62 to 81% [4, 13, 18].

It is tempting to speculate that proven approaches and even promising experimental therapies for severely injured trauma patients can also play a role in the management of critically ill COVID-19 patients. This review will compare pathophysiology and treatment strategies between severe trauma and life-threatening SARS-CoV-2 infections.

### Pathophysiology of severe trauma

In trauma, tissue damage activates the immune response, with extensive tissue damage invoking systemic inflammation [6, 15, 16]. Alterations in local and systemic immune responses after severe trauma are recognized as a physiological reaction to restore homeostasis. The magnitude of these immunological changes correlates with the degree of local and systemic tissue damage [6, 19].

Necrotic cells rapidly release alarmins (Damage Associated Molecular Patterns (DAMPs)), which are endogenous molecules and are equivalent in function to pathogen associated molecular patterns (PAMPs, e.g. SARS-CoV-2 proteins). As their name suggests, alarmins alert the immune system and their ultimate function is to restore homeostasis by promoting regeneration of damaged tissue [6, 16, 20, 21].

In trauma, various relevant alarmins have been identified and characterized. One such alarmin, High Mobility Group Box 1 (HMGB1), has shown chemotactic effects on monocytes, macrophages, and neutrophils, and is a very potent stimulator of immune cell maturation [22]. In addition, Heat Shock Protein (HSP) interacts with several receptors including toll-like receptors and stimulate the secretion of proinflammatory cytokines such as tumor necrosis factor (TNF)-α and interleukin (IL)-1β [23]. These cytokines are early regulators of the pro-inflammatory immune response to trauma, and both of them induce the release of secondary cytokines, such as IL-6 and IL-8 [6, 24]. These cytokines, which are predominantly produced by monocytes and macrophages, mediate a variety of frequently overlapping effects, and their actions can be additive [6, 24]. DAMPS and cytokines further activate different immune cells including neutrophils and monocytes via DAMP-receptors [25].

The balance and interplay of these different endogenous molecules dictates the clinical course in trauma patients. Overexpression of either pro-inflammatory or anti-inflammatory mediators may induce organ dysfunction. Whereas a predominantly pro-inflammatory response leads to the systemic inflammatory response syndrome (SIRS), a predominantly anti-inflammatory reaction leads to the compensatory anti-inflammatory response syndrome (CARS). In the absence of inflammatory complications, concurrent SIRS/CARS responses should be considered a physiological process and balance each other out. However, excessive SIRS can result in immune overreaction, while CARS may lead to immune suppression or paralysis, with a subsequent increased risk of infectious complications [6, 16, 20, 21]. Interestingly, in severe blunt trauma, it has been demonstrated that the early immune response is consistent with simultaneously increased expression of both pro-inflammatory genes (SIRS-response) and anti-inflammatory genes (CARS-response) [26]. Together, this response induces an increase in circulating cytokines resulting in a cytokine storm [27]. Moreover, the development of systemic inflammatory complications, such as organ failure, are associated with the magnitude and duration of a genomically induced cytokine storm [20, 21, 24, 26, 27].

Extrapulmonary inflammatory processes affect the pulmonary compartment via the circulatory uptake of these cytokines [6, 28]. One crucial step in the pathophysiology of distant organ damage is the adherence of activated polymorphonuclear leukocytes to capillary endothelial cells [29]). This is characterized by leukocyte diapedesis in organ tissue, with subsequent release of oxygen radicals and proteases [30]. These consequently damage the endothelial layer, resulting in increased capillary permeability, interstitial oedema and finally distant organ damage [6, 9, 16, 30]. Further inflammatory activation causes local collateral damage to parenchymal cells and result in subsequent lung dysfunction [6, 15, 16, 31]. The collaboration of humoral immune factors and immune cells play an essential role in the transition from an appropriate to an overwhelming, dysregulated immune response [6, 15, 16, 31, 32].

These pathophysiological inflammatory cascades result in significant changes in the anatomy, mechanics and function of the lung [33]. An initial increase in pulmonary capillary permeability stimulates alveolar flooding and oedema. The consequence is the loss of surfactant function. This causes atelectasis and subsequent alveolar instability, resulting in repetitive alveolar collapse and expansion, and finally impaired gas exchange with local and systemic hypoxia [32, 33].

### Pathophysiology of severe COVID-19

The exact pathophysiology of SARS-CoV-2 infection, and more specifically its considerable capacity to extensively disturb physiological homeostasis, is currently unclear. However, based on recent clinical and experimental findings and related viral diseases such as Severe Acute Respiratory Syndrome Coronavirus 1 (SARS-CoV-1), basic understanding has rapidly increased. The current SARS-CoV-2 virus, as well as the SARS-CoV-1 and the Middle East Respiratory Syndrome Coronavirus (MERS-CoV) are related pathological human coronaviruses (CoVs) [34–36] Phylogenetic studies show an 80% nucleotide identity of SARS-CoV-2 with SARS-CoV-1 and the pathophysiological insights made for SARS-CoV-1 can likely be applied to SARS-CoV-2 [37, 38].

In line with SARS-CoV-1, the angiotensin-converting enzyme 2 (ACE2), a type I membrane protein, is considered the host cell surface-receptor for the SARS-Cov-2 spike receptors [39, 40]. Kuba et al. demonstrated the key role of ACE2-receptors in SARS-CoV-1 pathology by showing that ACE2-KO mice were unsusceptible to SARS-CoV exposure [41]. Distribution of the ACE2-receptor is tissue specific, and varying degrees of receptor expression are seen in lungs (type I and type II alveolar epithelial cells as well as bronchiolar epithelial cells), heart, kidneys and the gastro-intestinal tract [42, 43]. Tissue specific ACE2-receptor distribution patterns may affect disease progression and make specific organs more susceptible. This is supported by histopathological studies of SARS-CoV fatalities showing most prominent tissue damage in the respiratory system with less extensive damage to liver, kidney, cardiac and digestive tract tissues [44, 45].

ACE2 plays an important physiological role in regulating blood pressure via the renin-angiotensin-aldosterone system (RAAS). It is therefore tempting to hypothesize that there is an interplay between clinical observations linking hypertension with impaired outcome in SARS-Cov-2 infection [46, 47]. Upon viral cell invasion, however, the process leading to end-organ parenchymal cell destruction and finally resulting in life threatening complications such as ARDS and MODS remain unclear. SARS-CoV-1 mice models demonstrated the relevance of ACE2-expression and suggests that SARS-CoV may directly derange ACE2-lung protective pathways [48].

In addition to these direct pathophysiological processes of SARS, it is likely that other indirect processes also play a critical role in the rapid progression of severe COVID-19. The SARS-CoV-1 epidemic emphasized this with the observation that disease progression into critical stages was commonly associated with diminishing viral titers [34, 49]. Studies on the association between viral load and COVID-19 disease severity, however, are scarce, and have so far demonstrated conflicting results [50, 51]. Post-mortem observations in SARS-CoV-1 and SARS-CoV-2 are characterized by alveolar exudative inflammation, interstitial inflammation, alveolar epithelial proliferation, and hyaline membrane formation, thereby demonstrating ARDS-like lesions [4, 5, 52–54].

From an immunological perspective, a prominent neutrophil and macrophage presence was observed in the pulmonary interstitium and alveoli in SARS-CoV-1 fatalities [54]. The aberrant systemic cellular immune response of fatal SARS-CoV-1-cases was characterized by both an increase in circulating monocytes and neutrophils and a reduction in circulating lymphocytes [55, 56].

In SARS-CoV-2, lymphopenia is common [4, 12] and both circulatory lymphopenia and leukocytosis are associated with COVID-19 mortality [10]. In contrast with mild cases of COVID-19, a reduction of circulatory CD4+ and CD8+ cells was observed in severe COVID-19 infection [57, 58].

In addition to an altered cellular immune response in fatal SARS-CoV-1/2 infections, transient humoral dysregulation has also been described. Whereas minimal cytokine level alterations are seen in mild COVID-19 infection [57], severe infections have shown elevated levels of pro-inflammatory cytokines including TNF-alpha, IL-6, IL-10 [58].

Although experimental clinical data on COVID-19 infection is scarce, the presence of an aberrant humoral immune response is further suggested by significantly increased serum IL-6 levels found in COVID-19 fatalities [59, 60]. Moreover, a cytokine storm, indicative of widespread immune activation similar to that found in posttraumatic ARDS, has also been observed in SARS-CoV-2 infection [26, 59–61].

**Fig. 1:**
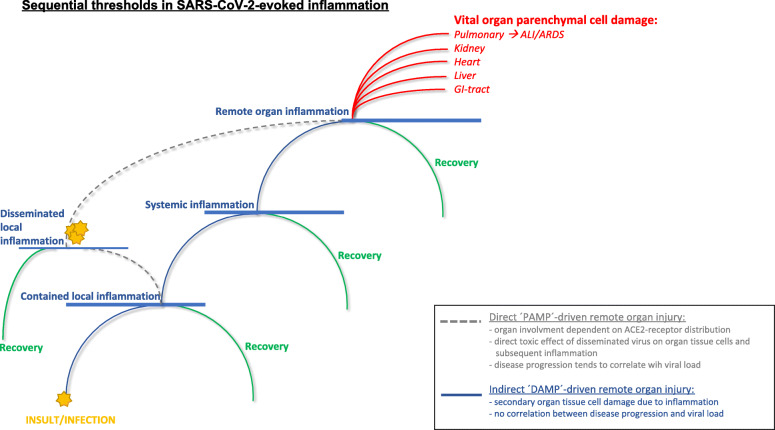
Sequential thresholds in virus-evoked inflammation. A hypothetical multifactorial model of disease progression in COVID-19 is presented. Two specific pathways have been described, and an interplay is likely to occur. At all phases restoration of homeostasis is possible and will lead to recovery. The development of differentiated treatment concepts may benefit from this model, guide tailored interventions at different stages of disease progression. *Abbreviations*: ALI, acute lung injury; *ARDS*, acute respiratory distress syndrome; *PAMPs,* pathogen associated molecular patterns; *DAMPs,* damage associated molecular patterns

Based on these findings, it is tempting to hypothesize that rather than direct cytotoxic effects of virus-infected cells alone, severe SARS-CoV-2 infection triggers both the humoral and cellular immune system. Consequent life-threatening complications therefore primarily relate to an aberrant immune response. A similar aberrant immune response after severe trauma has been well established. The pathophysiological mechanisms observed in trauma may be universal and are therefore likely also involved in COVID-19 disease progression. Insights in the pathophysiology of severe trauma may therefore form the basis for novel therapeutic concepts for SARS-CoV-2 and we propose the pathophysiological model of COVID-19 disease progression as being displayed as Fig. 1.

This figure summarizes the process of ongoing inflammation, starting from *insult* (viral infection), into the development of end-organ inflammation and dysfunction. The initial insult evokes a local systemic response including involvement of natural killer cell/dendritic cells, lymphocytes, neutrophils and macrophages. Adequate responses result in viral clearance and in the case of *contained local infection*, minimal collateral damage to parenchymal organ cells and consequent recovery. However, in case the immune response is unable to stop ongoing viral replication, disease progression may occur. Based on the pathophysiological concepts in SARS-CoV-1, SARS-CoV-2 and trauma summarized above, it is tempting to hypothesize that COVID-19 disease progression is based on two primary mechanistic pathways. These pathways are a direct (PAMP-driven) pathway and an indirect (DAMP-driven) pathway, either of which can lead to COVID-19 disease progression:

(i) A *direct (‘PAMP’)-driven pathway*. This is characterized by increased local spread of the virus and eventually *dissemination* of viral infection (dependent on tissue-specific ACE2-receptor expression levels and likely correlating with increased viral load). Vital organ involvement may occur, even in the absence of initial systemic inflammation, leading to (multiple) organ dysfunction.
(ii) An *indirect (‘DAMP’)-driven pathway*. Upon contained local inflammation, a secondary massive immune response may be initiated (including a cytokine storm, large-scale innate immune cell activation and SIRS/CARS-responses). These processes lead to extensive systemic inflammation and subsequent altered homeostasis of vital organs. Finally, organ failure may occur due to inflammation-induced collateral damage in end-organs. Progression according to this pathway is independent of viral load.

These are not mutually exclusive pathways, and interplay between these may potentially play a role in the progression of COVID-19. At all phases of both pathways, improvement and restoration of homeostasis is possible and will lead to recovery. 

### First and second hit mechanism in severe trauma and COVID-19

#### ‘First hit’

In traumatology, the term ‘first hit’ is used to describe the initial insult condition, or trauma, the patient faces. The intensity of the first hit is based on the trauma load: the degree of initial tissue damage, including organ injury, fractures, burn injury, soft tissue injury, and hypovolemic shock. Consequently, local and systemic release of both pro-inflammatory and anti-inflammatory mediators is dependent on the severity of the first hit of the trauma itself [62]. These insults determine the initial trauma load and result in activation of the innate and adaptive immune system that stimulate the local and systemic inflammatory reactions. Moreover, genetic predisposition seems to affect the immune response as well [6]. The subsequent activation of immune cells (polymorphonuclear granulocytes, monocytes and lymphocytes) trigger multifocal processes leading to tissue regeneration and repair. Excessive tissue damage, however, may magnify the extent of local and systemic activation leading to organ dysfunction. Whereas a predominantly pro-inflammatory response leads to an excessive SIRS-response, a predominantly anti-inflammatory reaction may result in an intensified CARS-response and with subsequent immunoparalysis and a consequent increased risk of infectious complications [6, 15, 16].

Similarly, with COVID-19, the first hit is the initial viral infection itself, centred primarily on respiratory tissue. The magnitude of the first hit is associated with predisposing factors (e.g. sex, expression of ACE2 receptors) and viral load. Existing comorbidities may also affect the severity of the initial insult and determine the overall impact of the first hit on homeostasis. Patients with diabetes, cardiovascular disease, hypertension and lung disease are especially prone to decompensation in SARS-CoV-2 infection [4, 12].

#### ‘Second hit’

In trauma cases, the secondary activation of various molecular cascades due to an additional insult are known as second hit- phenomena. This secondary immune cell activation, or priming, is stimulated by a variety of triggers [6, 15, 16, 31]. These triggers may be iatrogenic (e.g. mechanical ventilation [63], surgical intervention [20, 21], transfusions) or non-iatrogenic (e.g. secondary infection [64], thromboembolic complications [65], ischemia/reperfusion injury [66]. Each insult further catalyses the immune response, and depending on severity may cause an excessive inflammatory response. This process initiates a vicious cycle of local tissue damage, additionally aggravated by systemic hyperactivation ultimately leading to a life-threatening, overwhelming immune response.

**Fig. 2:**
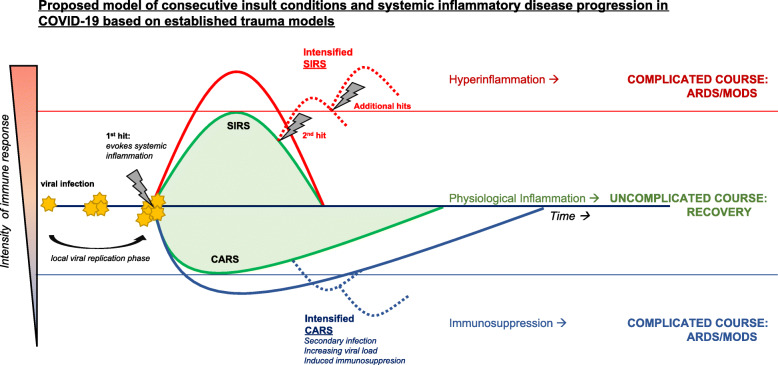
Proposed model of consecutive insult conditions and systemic inflammatory disease progression in COVID-19, based on established trauma modelling. Established concepts in the field of severe trauma and related inflammatory complications have been applied to COVID-19 disease progression. Systemic inflammation may contribute to the restoration of homeostasis and should be considered a physiological process. However, altered inflammatory response may lead to hyperinflammation or a pathological hypo-inflammatory immune response. See text for details and explanations. Abbreviations: SIRS, systemic inflammatory response syndrome; CARS, compensatory anti-inflammatory response syndrome

Similarly, in severe COVID-19 infection, secondary insults or complications may stimulate an exaggerated immune response, amplify systemic inflammation, or at a later stage induce immune paralysis. Respiratory distress and systemic hypoxemia may stimulate further tissue injury and abruptly require intensive care and monitoring. In addition, metabolic decompensation and systemic hypoperfusion are further risk factors associated with remote tissue damage. Secondary pulmonary bacterial infections or catheter-associated infections are further potential second hit events, all of which are capable of aggravating the clinical course of the disease. Figure 2 displays our proposed model of consecutive insults and systemic inflammatory disease progression in COVID-19, based on established trauma models. Like trauma, viral infection causes rapid activation of the immune system. In case the first hit insult is potent enough, systemic inflammation occurs. Upon initiation of systemic inflammation, both a systemic inflammatory response syndrome (SIRS) and a compensatory anti-inflammatory response syndrome (CARS) are evoked. The green curves in Fig. 2 represent uncomplicated courses and lead to recovery. In these courses, the inflammatory response is considered a physiological process.

In some patients, however, immune dysregulation occurs. Excessive immune activation (hyperactivation, *red curves in* Fig. 2) may occur, and collateral damage to parenchymal cells of vital organs may lead to organ failure or multiple organ dysfunction syndrome (MODS). Alternatively, patients may develop inflammatory complications due to immune paralysis as a result of CARS. A refractory state of the immune system is incapable of resolving the SARS-CoV-2 infection and are more susceptible to novel pathogens. Complications develop when either the primary viral or secondary microbial infection cannot be resolved. Eventually, infection-related MODS may occur (*blue curves in* Fig. 2). In both cases, additional hits may push a patient from a physiological immune response to either a pathological hyperinflammatory immune response or a pathological hypo-inflammatory immune response.

### Potential treatment strategies for severe COVID-19 disease

Numerous fast-tracked clinical trials to treat COVID-19 infections have recently been initiated. Many therapeutic strategies focus primarily on inhibiting the virus, or bolstering the immune system. These strategies are aimed at treating the direct PAMP-driven pathophysiological pathway, thereby supporting the immune response.

Based on experience treating ARDS in trauma, trying to do more may in fact be less. The majority of these experimental treatments may be focusing on the wrong enemy: the virus, instead of the hosts’ uncontrolled immune response. Alternatively, as in trauma, anti-inflammatory interventions to modulate the hyperactive immune response in COVID-19 may be promising. Most modern treatment modalities for trauma are aimed at the indirect DAMP-driven pathway, and to dampen second hit events. Applying these concepts to COVID-19 treatment, the following measures complementary to current treatment guidelines for COVID-19 disease should be considered:

1. postponing all elective, non-essential surgical interventions in endemic areas. This has already been adopted by many clinicians treating COVID-19 and should be universally adopted. Furthermore, execution of invasive diagnostic procedures and interventions should be limited to live-saving interventions only.
2. lung-protective ventilation protocols to prevent a barotrauma second hit [67]. Mechanical ventilation when improperly used can exacerbate lung damage by causing secondary ventilatory induced lung injury (VILI). VILI can be significantly reduced with proper positive end-expiratory pressure (PEEP) levels to minimize atelectasis [67]. Moreover, lowering tidal volume (Vt) and plateau pressure (Pplat) may prevent lung over-distension. Alveolar strain can be decreased by reducing the transpulmonary pressure (Ptp) gradient [68].
3. sufficient thrombosis prophylaxis to prevent thromboembolic second hits. In cases where medicinal prophylaxis is contraindicated, mechanical measures including compression stockings or intermittent pneumatic compression should be considered. Further, several studies have described a close link between thrombogenesis and inflammation. Proinflammatory cytokines (e.g. IL-6) stimulate the expression of prothrombotic mediators. Dampening the proinflammatory immune response may further minimize the risk of thromboembolic complications [69]. Increased serum D-dimer levels in severe cases of COVID-19 and the frequent occurrence of embolic complications underline the relevance of impaired thromboembolic homeostasis in the specific case of COVID-19 [70].
4. transfusion of blood products should be minimized to avoid transfusion induced immune activation and more specifically transfusion-related acute injury (TRALI)-like conditions [71].
5. prevent and treat secondary infections adequately to prevent additional infectious insults and inflammatory exaggeration. We suggest actively searching for secondary infections by serial clinical examination and routine laboratory analysis of infection parameters. Additionally, catheter associated infections can be minimized with regular renewal schedules of catheters [72].

Table 1 provides an overview of standard measures used to optimize care of critically ill trauma patients that can be applied to severe SARS-CoV-2 infections. As a next step, experimental immunomodulatory therapies aimed to optimize outcomes of trauma induced inflammatory complications may be considered for the treatment of SARS-CoV-2. Potential therapies include tissue plasminogen activator (tPA) [73], the anti-inflammatory effects of tranexamic acid [74], and extracorporeal cytokine adsorption therapy [75].

**Table 1:** Overview of insult conditions in severe trauma vs. severe COVID-19 and suggested therapeutic targets

Insult condition	*Severe trauma*	*Severe COVID-19*	*Targets for therapy*
1st hit *Evokes SIRS/CARS-response*	Initial trauma	Initial infection	Immunomodulation: Optimizes direct pathway Optimizes indirect pathway Avoid intensifying the immune response with therapeutic concepts aimed to ‘boost’ the immune response
2nd hits *Intensifies SIRS/CARS-response*	Surgery/ Invasive procedures	Invasive procedures	Minimizing interventions Postponing elective surgery
	Barotrauma (mechanical ventilation)	Barotrauma (mechanical ventilation)	Lung protective ventilation: - Avoiding high-PEEP - Lower tidal volume - Lower plateau pressure - Frequent prone positioning
	Thrombo-embolic events	Thrombo-embolic events	Adequate thrombosis prophylaxis: - Medication - Intermittent pneumatic compression - Automatic lateral rotation therapy - Early mobilization
	Transfusion of blood products	Transfusion of blood products	Minimizing transfusion Consider Tranexamic acid
	Secondary bacterial infection	Secondary bacterial infection	- Calculated anti-infective therapies - Frequent clinical examination to identify concurrent infection - Frequent determination of serum inflammatory markers - Routine renewal of urine and central catheters

## Conclusion

This review attempts to draw analogies to pathophysiological and therapeutic principles between severely injured trauma patients and COVID-19. Many similarities between both conditions have been identified, supporting the hypothesis that treatment concepts for ARDS in trauma may potentially be of use in the management of COVID-19. Two complementary pathways of disease progression into severe COVID-19 have been identified and described. Based on established pathophysiological concepts in the field of trauma, we strongly suggest postponing all elective surgery in endemic areas. Immunoprotective protocols such as lung protective ventilation, adequate thrombosis prophylaxis, prevention of secondary infection, and calculated antibiotic therapies used to minimize inflammatory complications in trauma patients are likely beneficial in the management of SARS-CoV-2 infections, and should be universally applied whenever possible. Finally, experimental immunomodulatory interventions currently being investigated in the setting of severe trauma may play a role in the management of COVID-19 patients and should be investigated.

## Data Availability

Data sharing is not applicable to this article as no datasets were generated or analysed during the current study.

## Abbreviations

SARS-CoV-2: Severe acute respiratory syndrome coronavirus 2
ARDS: Acute respiratory distress syndrome
COVID-19: Corona virus disease 2019
MODS: Multiple organ dysfunction syndrome
ICU: Intensive care unit
DAMPs: Damage associated molecular patterns
PAMPs: pathogen associated molecular patterns
HMGB1: High mobility group box 1
HSP: Heat shock protein
TNF: Tumor necrosis factor’
IL: Interleukin
SIRS: Systemic inflammatory response syndrome
CARS: Compensatory anti-inflammatory response syndrome
SARS-CoV-1: Severe acute respiratory syndrome coronavirus 1
MERS-CoV: Middle east respiratory syndrome coronavirus
CoVs: Coronaviruses
ACE2: Angiotensin-converting enzyme 2
RAAS: Renin-angiotensinaldosterone system
VILI: Ventilatory induced lung injury
PEEP: Positive end-expiratory pressure
Vt: Tidal volume
Pplat: Plateau pressure
Ptp: Transpulmonary pressure
TRALI: Transfusion-related acute injury
tPA: Tissue plasminogen activator

## References

[1] Zhou P, Yang XL, Wang XG, Hu B, Zhang L, Zhang W, Si HR, Zhu Y, Li B, Huang CL, Chen HD, Chen J, Luo Y, Guo H, Jiang RD, Liu MQ, Chen Y, Shen XR, Wang X, Zheng XS, Zhao K, Chen QJ, Deng F, Liu LL, Yan B, Zhan FX, Wang YY, Xiao GF, Shi ZL (2020). A pneumonia outbreak associated with a new coronavirus of probable bat origin. Nature..

[2] World Health Organisation: WHO Director-General’s opening remarks at the media briefing on COVID-19. https://www.who.int/dg/speeches/detail/who-director-general-s-opening-remarks-at-the-media-briefing-on-covid-19%2D%2D-20-march-2020. Accessed 3 May 2020.

[3] Ranney ML, Griffeth V, Jha AK (2020). Critical supply shortages - the need for ventilators and personal protective equipment during the Covid-19 pandemic. N Engl J Med.

[4] Yang X, Yu Y, Xu J, Shu H, Xia J, Liu H, Wu Y, Zhang L, Yu Z, Fang M, Yu T, Wang Y, Pan S, Zou X, Yuan S, Shang Y (2020). Clinical course and outcomes of critically ill patients with SARS-CoV-2 pneumonia in Wuhan, China: a single-centered, retrospective, observational study. Lancet Respir Med.

[5] Xu Z, Shi L, Wang Y, Zhang J, Huang L, Zhang C, Liu S, Zhao P, Liu H, Zhu L, Tai Y, Bai C, Gao T, Song J, Xia P, Dong J, Zhao J, Wang FS (2020). Pathological findings of COVID-19 associated with acute respiratory distress syndrome. Lancet Respir Med.

[6] Keel M, Trentz O (2005). Pathophysiology of polytrauma. Injury..

[7] Watkins TR, Nathens AB, Cooke CR, Psaty BM, Maier RV, Cuschieri J, Rubenfeld GD (2012). Acute respiratory distress syndrome after trauma: development and validation of a predictive model. Crit Care Med.

[8] Pfeifer R, Heussen N, Michalewicz E, Hilgers RD, Pape HC (2017). Incidence of adult respiratory distress syndrome in trauma patients: a systematic review and meta-analysis over a period of three decades. J Trauma Acute Care Surg.

[9] Huang C, Wang Y, Li X, Ren L, Zhao J, Hu Y, Zhang L, Fan G, Xu J, Gu X, Cheng Z, Yu T, Xia J, Wei Y, Wu W, Xie X, Yin W, Li H, Liu M, Xiao Y, Gao H, Guo L, Xie J, Wang G, Jiang R, Gao Z, Jin Q, Wang J, Cao B (2020). Clinical features of patients infected with 2019 novel coronavirus in Wuhan, China. Lancet.

[10] Zhou F, Yu T, Du R, Fan G, Liu Y, Liu Z, Xiang J, Wang Y, Song B, Gu X, Guan L, Wei Y, Li H, Wu X, Xu J, Tu S, Zhang Y, Chen H, Cao B (2020). Clinical course and risk factors for mortality of adult inpatients with COVID-19 in Wuhan, China: a retrospective cohort study. Lancet..

[11] Wu C, Chen X, Cai Y, Xia J, Zhou X, Xu S, Huang H, Zhang L, Zhou X, Du C, Zhang Y, Song J, Wang S, Chao Y, Yang Z, Xu J, Zhou X, Chen D, Xiong W, Xu L, Zhou F, Jiang J, Bai C, Zheng J, Song Y. Risk Factors Associated With Acute Respiratory Distress Syndrome and Death in Patients With Coronavirus Disease 2019 Pneumonia in Wuhan, China. JAMA Intern Med. 2020:e200994. 10.1001/jamainternmed.2020.0994. 10.1001/jamainternmed.2020.0994 PMC7070509 32167524

[12] Guan WJ, Ni ZY, Hu Y, Liang WH, Ou CQ, He JX, Liu L, Shan H, Lei CL, Hui DSC, Du B, Li LJ, Zeng G, Yuen KY, Chen RC, Tang CL, Wang T, Chen PY, Xiang J, Li SY, Wang JL, Liang ZJ, Peng YX, Wei L, Liu Y, Hu YH, Peng P, Wang JM, Liu JY, Chen Z, Li G, Zheng ZJ, Qiu SQ, Luo J, Ye CJ, Zhu SY, Zhong NS (2020). China medical treatment expert Group for Covid-19. Clinical characteristics of coronavirus disease 2019 in China. N Engl J Med.

[13] Verity R, Okell LC, Dorigatti I, Winskill P, Whittaker C, Imai N, Cuomo-Dannenburg G, Thompson H, Walker PGT, Fu H, Dighe A, Griffin JT, Baguelin M, Bhatia S, Boonyasiri A, Cori A, Cucunubá Z, FitzJohn R, Gaythorpe K, Green W, Hamlet A, Hinsley W, Laydon D, Nedjati-Gilani G, Riley S, van Elsland S, Volz E, Wang H, Wang Y, Xi X, Donnelly CA, Ghani AC, Ferguson NM (2020). Estimates of the severity of coronavirus disease 2019: a model-based analysis. Lancet Infect Dis.

[14] Pfeifer R, Tarkin IS, Rocos B, Pape HC (2009). Patterns of mortality and causes of death in polytrauma patients--has anything changed?. Injury..

[15] Lord JM, Midwinter MJ, Chen YF, Belli A, Brohi K, Kovacs EJ, Koenderman L, Kubes P, Lilford RJ (2014). The systemic immune response to trauma: an overview of pathophysiology and treatment. Lancet..

[16] Griensven van M, Krettek C, Pape HC (2003). Immune reactions after trauma. Eur J Trauma.

[17] Pugin J (2012). How tissue injury alarms the immune system and causes a systemic inflammatory response syndrome. Ann Intensive Care.

[18] Wang D, Hu B, Hu C, Zhu F, Liu X, Zhang J, Wang B, Xiang H, Cheng Z, Xiong Y, Zhao Y, Li Y, Wang X, Peng Z (2020). Clinical characteristics of 138 hospitalized patients with 2019 novel coronavirus-infected pneumonia in Wuhan, China. JAMA.

[19] Okeny PK, Ongom P, Kituuka O (2015). Serum interleukin-6 level as an early marker of injury severity in trauma patients in an urban low-income setting: a cross-sectional study. BMC Emerg Med.

[20] Pape HC, Schmidt RE, Rice J, van Griensven M, das Gupta R, Krettek C, Tscherne H (2000). Biochemical changes after trauma and skeletal surgery of the lower extremity: quantification of the operative burden. Crit Care Med.

[21] Pape HC, Grimme K, Van Griensven M, Sott AH, Giannoudis P, Morley J, Roise O, Ellingsen E, Hildebrand F, Wiese B, Krettek C, EPOFF Study Group (2003). Impact of intramedullary instrumentation versus damage control for femoral fractures on immunoinflammatory parameters: prospective randomized analysis by the EPOFF study group. J Trauma.

[22] Cohen MJ, Brohi K, Calfee CS, Rahn P, Chesebro BB, Christiaans SC, Carles M, Howard M, Pittet JF (2009). Early release of high mobility group box nuclear protein 1 after severe trauma in humans: role of injury severity and tissue hypoperfusion. Crit Care.

[23] Zhang Z, Zhang ZY, Wu Y, Schluesener HJ (2012). Immunolocalization of toll-like receptors 2 and 4 as well as their endogenous ligand, heat shock protein 70, in rat traumatic brain injury. Neuroimmunomodulation..

[24] Hildebrand F, Pape HC, Krettek C (2005). The importance of cytokines in the posttraumatic inflammatory reaction. Unfallchirurg..

[25] Prince LR, Whyte MK, Sabroe I, Parker LC (2011). The role of TLRs in neutrophil activation. Curr Opin Pharmacol.

[26] Xiao W, Mindrinos MN, Seok J, Cuschieri J, Cuenca AG, Gao H, Hayden DL, Hennessy L, Moore EE, Minei JP, Bankey PE, Johnson JL, Sperry J, Nathens AB, Billiar TR, West MA, Brownstein BH, Mason PH, Baker HV, Finnerty CC, Jeschke MG, López MC, Klein MB, Gamelli RL, Gibran NS, Arnoldo B, Xu W, Zhang Y, Calvano SE, GP MD-S, Schoenfeld DA, Storey JD, Cobb JP, Warren HS, Moldawer LL, Herndon DN, Lowry SF, Maier RV, Davis RW, Tompkins RG (2011). Inflammation and Host Response to Injury Large-Scale Collaborative Research Program. A genomic storm in critically injured humans. J Exp Med.

[27] Clark IA, Vissel B (2017). The meteorology of cytokine storms, and the clinical usefulness of this knowledge. Semin Immunopathol.

[28] Hudson LD, Milberg JA, Anardi D, Maunder RJ (1995). Clinical risks for development of the acute respiratory distress syndrome. Am J Respir Crit Care Med.

[29] Eppihimer MJ, Granger DN (1997). Ischemia/reperfusion-induced leukocyte-endothelial interactions in postcapillary venules. Shock..

[30] Roumen RM, Hendriks T, van der Ven-Jongekrijg J (1993). Cytokine patterns in patients after major vascular surgery, hemorrhagic shock, and severe blunt trauma. Relation with subsequent adult respiratory distress syndrome and multiple organ failure. Ann Surg.

[31] Adams JM, Hauser CJ, Livingston DH, Lavery RF, Fekete Z, Deitch EA (2001). Early trauma polymorphonuclear neutrophil responses to chemokines are associated with development of sepsis, pneumonia, and organ failure. J Trauma.

[32] Nieman G, Satalin J, Andrews P, Wilcox K, Aiash H, Baker S, Kollisch-Singule M, Madden M, Gatto L, Habashi N (2018). Preemptive mechanical ventilation based on dynamic physiology in the alveolar microenvironment: novel considerations of time-dependent properties of the respiratory system. J Trauma Acute Care Surg.

[33] Bellingan GJ (2002). The pulmonary physician in critical care 6: the pathogenesis of ALI/ARDS. Thorax..

[34] Channappanavar R, Perlman S (2017). Pathogenic human coronavirus infections: causes and consequences of cytokine storm and immunopathology. Semin Immunopathol.

[35] Perlman S, Netland J (2009). Coronaviruses post-SARS: update on replication and pathogenesis. Nat Rev Microbiol.

[36] Alagaili AN, Briese T, Mishra N, Kapoor V, Sameroff SC, Burbelo PD, de Wit E, Munster VJ, Hensley LE, Zalmout IS, Kapoor A, Epstein JH, Karesh WB, Daszak P, Mohammed OB, Lipkin WI (2014). Middle East respiratory syndrome coronavirus infection in dromedary camels in Saudi Arabia. mBio.

[37] Tang X, Wu C, Li X, Song Y, Yao X, Wu X, Duan Y, Zhang H, Wang Y, Qian Z, Cui J, Lu J. On the origin and continuing evolution of SARS-CoV-2. Natl Sci Rev. 2020. 10.1093/nsr/nwaa036. 10.1093/nsr/nwaa036 PMC7107875 34676127

[38] Gralinski LE, Menachery VD (2020). Return of the coronavirus: 2019-nCoV. Viruses..

[39] Hoffmann M, Kleine-Weber H, Schroeder S, Krüger N, Herrler T, Erichsen S, Schiergens TS, Herrler G, Wu NH, Nitsche A, Müller MA, Drosten C, Pöhlmann S (2020). SARS-CoV-2 Cell Entry Depends on ACE2 and TMPRSS2 and Is Blocked by a Clinically Proven Protease Inhibitor. Cell.

[40] He L, Ding Y, Zhang Q, Che X, He Y, Shen H, Wang H, Li Z, Zhao L, Geng J, Deng Y, Yang L, Li J, Cai J, Qiu L, Wen K, Xu X, Jiang S (2006). Expression of elevated levels of pro-inflammatory cytokines in SARS-CoV-infected ACE2+ cells in SARS patients: relation to the acute lung injury and pathogenesis of SARS. J Pathol.

[41] Kuba K, Imai Y, Rao S, Gao H, Guo F, Guan B, Huan Y, Yang P, Zhang Y, Deng W, Bao L, Zhang B, Liu G, Wang Z, Chappell M, Liu Y, Zheng D, Leibbrandt A, Wada T, Slutsky AS, Liu D, Qin C, Jiang C, Penninger JM (2005). A crucial role of angiotensin converting enzyme 2 (ACE2) in SARS coronavirus-induced lung injury. Nat Med.

[42] Hamming I, Timens W, Bulthuis ML, Lely AT, Navis G, van Goor H (2004). Tissue distribution of ACE2 protein, the functional receptor for SARS coronavirus. A first step in understanding SARS pathogenesis. J Pathol.

[43] Zhao Y, Zhao Z, Wang Y, et al. Single-cell RNA expression profiling of ACE2, the putative receptor of Wuhan2019-nCOv. bioRxic. 2020. 10.1101/2020.01.26.919985.

[44] Ding Y, He L, Zhang Q, Huang Z, Che X, Hou J, Wang H, Shen H, Qiu L, Li Z, Geng J, Cai J, Han H, Li X, Kang W, Weng D, Liang P, Jiang S (2004). Organ distribution of severe acute respiratory syndrome (SARS) associated coronavirus (SARS-CoV) in SARS patients: implications for pathogenesis and virus transmission pathways. J Pathol.

[45] Gu J, Gong E, Zhang B, Zheng J, Gao Z, Zhong Y, Zou W, Zhan J, Wang S, Xie Z, Zhuang H, Wu B, Zhong H, Shao H, Fang W, Gao D, Pei F, Li X, He Z, Xu D, Shi X, Anderson VM, Leong AS (2005). Multiple organ infection and the pathogenesis of SARS. J Exp Med.

[46] Li B, Yang J, Zhao F, Zhi L, Wang X, Liu L, Bi Z, Zhao Y (2020). Prevalence and impact of cardiovascular metabolic diseases on COVID-19 in China. Clin Res Cardiol.

[47] Donoghue M, Hsieh F, Baronas E, Godbout K, Gosselin M, Stagliano N, Donovan M, Woolf B, Robison K, Jeyaseelan R, Breitbart RE, Acton S (2000). A novel angiotensin-converting enzyme-related carboxypeptidase (ACE2) converts angiotensin I to angiotensin 1-9. Circ Res.

[48] Imai Y, Kuba K, Rao S, Huan Y, Guo F, Guan B, Yang P, Sarao R, Wada T, Leong-Poi H, Crackower MA, Fukamizu A, Hui CC, Hein L, Uhlig S, Slutsky AS, Jiang C, Penninger JM (2005). Angiotensin-converting enzyme 2 protects from severe acute lung failure. Nature..

[49] Peiris JS, Chu CM, Cheng VC, Chan KS, Hung IF, Poon LL, Law KI, Tang BS, Hon TY, Chan CS, Chan KH, Ng JS, Zheng BJ, Ng WL, Lai RW, Guan Y, Yuen KY, HKU/UCH SARS study group (2003). Clinical progression and viral load in a community outbreak of coronavirus-associated SARS pneumonia: a prospective study. Lancet..

[50] Tsang OT, Leung WS, Tam AR, Wu TC, Lung DC, Yip CC, Cai JP, Chan JM, Chik TS, Lau DP, Choi CY, Chen LL, Chan WM, Chan KH, Ip JD, Ng AC, Poon RW, Luo CT, Cheng VC, Chan JF, Hung IF, Chen Z, Chen H, Yuen KY, To KK (2020). Temporal profiles of viral load in posterior oropharyngeal saliva samples and serum antibody responses during infection by SARS-CoV-2: an observational cohort study. Lancet Infect Dis.

[51] Liu Y, Yan LM, Wan L, Xiang TX, Le A, Liu JM, Peiris M, Poon LLM, Zhang W (2020). Viral dynamics in mild and severe cases of COVID-19. Lancet Infect Dis.

[52] Liu J, Zheng X, Tong Q, Li W, Wang B, Sutter K, Trilling M, Lu M, Dittmer U, Yang D (2020). Overlapping and discrete aspects of the pathology and pathogenesis of the emerging human pathogenic coronaviruses SARS-CoV, MERS-CoV, and 2019-nCoV. J Med Virol.

[53] Yao XH, Li TY, He ZC, Ping YF, Liu HW, Yu SC, Mou HM, Wang LH, Zhang HR, Fu WJ, Luo T, Liu F, Guo QN, Chen C, Xiao HL, Guo HT, Lin S, Xiang DF, Shi Y, Pan GQ, Li QR, Huang X, Cui Y, Liu XZ, Tang W, Pan PF, Huang XQ, Ding YQ, Bian XW (2020). A pathological report of three COVID-19 cases by minimal invasive autopsies. Zhonghua Bing Li Xue Za Zhi.

[54] Nicholls JM, Poon LL, Lee KC, Ng WF, Lai ST, Leung CY, Chu CM, Hui PK, Mak KL, Lim W, Yan KW, Chan KH, Tsang NC, Guan Y, Yuen KY, Peiris JS (2003). Lung pathology of fatal severe acute respiratory syndrome. Lancet..

[55] Cui W, Fan Y, Wu W, Zhang F, Wang JY, Ni AP (2003). Expression of lymphocytes and lymphocyte subsets in patients with severe acute respiratory syndrome. Clin Infect Dis.

[56] Wang YH, Lin AS, Chao TY, Lu SN, Liu JW, Chen SS, Lin MC (2004). A cluster of patients with severe acute respiratory syndrome in a chest ward in southern Taiwan. Intensive Care Med.

[57] Thevarajan I, Nguyen THO, Koutsakos M, Druce J, Caly L, van de Sandt CE, Jia X, Nicholson S, Catton M, Cowie B, Tong SYC, Lewin SR, Kedzierska K (2020). Breadth of concomitant immune responses prior to patient recovery: a case report of non-severe COVID-19. Nat Med.

[58] Diao B, Wang C, Tan Y, Chen X, Liu Y, Ning L, Chen L, Li M, Liu Y, Wang G, Yuan Z, Feng Z, Zhang Y, Wu Y, Chen Y (2020). Reduction and functional exhaustion of T cells in patients with coronavirus disease 2019 (COVID-19). Front Immunol.

[59] Wang W Jr, He J, Lie P, et al. The definition and risks of cytokine release syndrome-like in 11 COVID-19-infected pneumonia critically ill patients: disease characteristics and retrospective analysis. MedRxic. 2020. 10.1101/2020.02.26.20026989.

[60] Ruan Q, Yang K, Wang W, Jiang L, Song J (2020). Clinical predictors of mortality due to COVID-19 based on an analysis of data of 150 patients from Wuhan, China. Intensive Care Med.

[61] Chen C, Zhang XR, Ju ZY, He WF (2020). Advances in the research of cytokine storm mechanism induced by Corona virus disease 2019 and the corresponding immunotherapies. Zhonghua Shao Shang Za Zhi.

[62] Moore FA, Sauaia A, Moore EE, Haenel JB, Burch JM, Lezotte DC (1996). Postinjury multiple organ failure: a bimodal phenomenon. J Trauma.

[63] van Wessem KJ, Hennus MP, Heeres M, Koenderman L, Leenen LP (2013). Mechanical ventilation is the determining factor in inducing an inflammatory response in a hemorrhagic shock model. J Surg Res.

[64] Vicente DA, Bradley MJ, Bograd B, Leonhardt C, Elster EA, Davis TA (2018). The impact of septic stimuli on the systemic inflammatory response and physiologic insult in a preclinical non-human primate model of polytraumatic injury. J Inflamm (Lond).

[65] Mukhopadhyay S, Johnson TA, Duru N, Buzza MS, Pawar NR, Sarkar R, Antalis TM (2019). Fibrinolysis and inflammation in venous Thrombus resolution. Front Immunol.

[66] van Hout GP, Teuben MP, Heeres M, de Maat S, de Jong R, Maas C, Kouwenberg LH, Koenderman L, van Solinge WW, de Jager SC, Pasterkamp G, Hoefer IE (2015). Invasive surgery reduces infarct size and preserves cardiac function in a porcine model of myocardial infarction. J Cell Mol Med.

[67] Petrucci N, De Feo C (2013). Lung protective ventilation strategy for the acute respiratory distress syndrome. Cochrane Database Syst Rev.

[68] Jaswal DS, Leung JM, Sun J, Cui X, Li Y, Kern S, Welsh J, Natanson C, Eichacker PQ (2014). Tidal volume and plateau pressure use for acute lung injury from 2000 to present: a systematic literature review. Crit Care Med.

[69] Kaski JC, Arrebola-Moreno AL (2011). Inflammation and thrombosis in atrial fibrillation. Rev Esp Cardiol.

[70] Danzi GB, Loffi M, Galeazzi G, Gherbesi E (2020). Acute pulmonary embolism and COVID-19 pneumonia: a random association?. Eur Heart J.

[71] Roubinian N (2018). TACO and TRALI: biology, risk factors, and prevention strategies. Hematology Am Soc Hematol Educ Program.

[72] Webster J, Osborne S, Rickard CM, New K (2015). Clinically-indicated replacement versus routine replacement of peripheral venous catheters. Cochrane Database Syst Rev.

[73] Moore HB, Barrett CD, Moore EE, McIntyre RC, Moore PK, Talmor DS, Moore FA, Yaffe MB (2020). Is there a role for tissue plasminogen activator as a novel treatment for refractory COVID-19 associated acute respiratory distress syndrome?. J Trauma Acute Care Surg.

[74] Teng Y, Feng C, Liu Y, Jin H, Gao Y, Li T (2018). Anti-inflammatory effect of tranexamic acid against trauma-hemorrhagic shock-induced acute lung injury in rats. Exp Anim.

[75] Schädler D, Pausch C, Heise D, Meier-Hellmann A, Brederlau J, Weiler N, Marx G, Putensen C, Spies C, Jörres A, Quintel M, Engel C, Kellum JA, Kuhlmann MK (2017). The effect of a novel extracorporeal cytokine hemoadsorption device on IL-6 elimination in septic patients: a randomized controlled trial. PLoS One.

